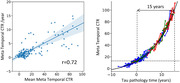# Unraveling the Early Trajectory of Cortical Tau Accumulation Using 18F‐MK6240

**DOI:** 10.1002/alz.093895

**Published:** 2025-01-09

**Authors:** Vincent Dore, Natasha Krishnadas, Pierrick Bourgeat, Antoine Leuzy, Azadeh Feizpour, Timothy Cox, Kun Huang, Samantha Budd Haeberlein, Jurgen Fripp, Victor L Villemagne, Christopher C. Rowe

**Affiliations:** ^1^ Department of Molecular Imaging & Therapy, Austin Health, Heidelberg, VIC Australia; ^2^ Florey Department of Neuroscience and Mental Health, The University of Melbourne, Heidelberg, VIC Australia; ^3^ The Australian e‐Health Research Centre, Commonwealth Scientific and Industrial Research Organisation, Brisbane, QLD Australia; ^4^ Clinical Memory Research Unit, Lund University, Lund, Skåne Sweden; ^5^ The Australian e‐Health Research Centre, Commonwealth Scientific and Industrial Research Organisation, Melbourne, VIC Australia; ^6^ Austin Health, Melbourne, VIC Australia; ^7^ Enigma, Toronto, ON Canada; ^8^ Austin Health, Heidelberg, VIC Australia

## Abstract

**Background:**

Tau PET is instrumental in tracking the longitudinal progression of Alzheimer’s disease (AD). 18F‐MK6240 is a high affinity tracer targeting the 3R/4R paired helical filaments of tau in AD. We aimed to evaluate the early phase of the natural progression of tau accumulation using 18F‐MK6240.

**Method:**

231 participants: 100 cognitively unimpaired (CU) Aß‐ (Centiloid<25CL), 58 CU Aß+, 73 cognitively impaired Aß+ (41 with mild cognitive impairment (MCI) and 32 with dementia) from the AIBL cohort were followed‐up with 18F‐MK6240 PET over one to four years (median 2.2years). Meta‐Temporal CenTauR (CTR) were generated using CapAIBL and CU CL<15, N = 120 and AD (typical AD tau pattern, MMSE>24, CL>50 & age<75, N = 39) as 0 and 100CTR anchored points. Abnormal level of tau was defined at 2 standard deviations above the CU Aß‐ (13CTR). Linear ordinary differential equations (ODE) were employed to model the mean natural history of CTR based on tau accumulators (CTR>13 or CTR rate>0) only. Given the limited numbers of individuals with CTR>100, our analysis concentrated on the early phase of tau accumulation (CTR<100, N = 204).

**Result:**

Figure1A illustrates a linear relationship between the average and the rate of CTR, with a R of 0.72. Tau accumulation spanned from 0.5CTR/yr at 13CTR to 12.2CTR/yr at 100CTR, with a standard deviation of the residuals at 2.4CTR/yr. Figure1B displays the individual’s trajectories projected on the ODE model. We estimated that, on average it takes 15.1 (CI:[12.6‐17.9]) years for an individual crossing 13CTR to reach 100CTR.

**Conclusion:**

Longitudinal 18F‐MK6240 is a robust tool for estimating natural progression of tau accumulation. Our findings indicate that it typically takes around 15 years to reach the tau levels associated with mild AD once tau starts aggregating in the neocortex. These findings shed light into the initial stages of cortical tau accumulation, relevant for early diagnosis and therapeutic interventions in AD.